# *In Vitro* Antiprotozoal Activity of Triterpenoid Constituents of *Kleinia odora* Growing in Saudi Arabia

**DOI:** 10.3390/molecules18089207

**Published:** 2013-07-31

**Authors:** Nawal M. Al Musayeib, Ramzi A. Mothana, Ali A. El Gamal, Shaza M. Al-Massarani, Louis Maes

**Affiliations:** 1 Department of Pharmacognosy, College of Pharmacy, King Saud University, Riyadh 11451, Saudi Arabia; E-Mails: aelgamal00@yahoo.com (A.A.-G.); salmassarani@ksu.edu.sa (S.M.-M.); 2 Department of Pharmacognosy, Faculty of Pharmacy, Sana’a University, P.O. Box 33039, Sana’a, Yemen; E-Mail: rmothana@ksu.edu.sa; 3 Laboratory for Microbiology, Parasitology and Hygiene (LMPH), Faculty of Pharmaceutical, Biomedical and Veterinary Sciences, Antwerp University, Antwerp B-2610, Belgium; E-Mail: louis.maes@ua.ac.be

**Keywords:** *Kleinia odora*, triterpenes, antiplasmodial, antileishmanial, antitrypanosomal

## Abstract

Two lupane and four ursane triterpenes, namely epilupeol (**1**), lupeol acetate (**2**), ursolic acid (**3**), brein (**4**), 3β 11α-dihydroxy urs-12-ene (**5**) and ursolic acid lactone (**6**) were isolated from aerial parts of *Kleinia odora* and identified. Compounds **1** and **3**–**6** were isolated for the first time from *K. odora.* The triterpene constituents were investigated for antiprotozoal potential against erythrocytic schizonts of *Plasmodium falciparum*, intracellular amastigotes of *Leishmania infantum* and *Trypanosoma cruzi* and free trypomastigotes of *T. brucei*. Cytotoxicity was determined against MRC-5 fibroblasts to assess selectivity. The ursane triterpenes were found to be active against more than one type of the tested parasites, with the exception of compound **6**. This is also the first report on the occurrence of ursane type triterpenes in the genus *Kleinia* and their antiprotozoal potential against *P. falciparum*, *L. infantum*, *T. cruzi*, and *T. brucei*.

## 1. Introduction

Protozoal infections are a worldwide health problem, particularly in developing countries [1,2,3,4], and approximately 14% of the World population are at risk of infection. Various studies have been conducted on leishmaniasis, malaria, Chagas and sleeping sickness which are considered major killing diseases [5]. The drugs currently in use mostly lack adequate efficacy, are toxic or show other liabilities, such as the need for parenteral application or high cost [6]. This reflects the need to continue searching for new and better antiprotozoal drugs. Natural products may offer good sources of leads for new drug design and discovery.

The genus *Kleinia* is a flowering plant comprising 40 species that are distributed in Somalia, the Middle-East, Madagascar and India [7,8]. Three species of this genus are commonly distributed in the Southern regions in Saudi Arabia: *K. odora* (Forssk) DC, *K. deflersii* (O.Schwartz) and *K. pendula* (Forssk) Sch. Bip. [9,10,11]. Several species of *Kleinia* are known to be rich sources of oxygenated sesquiterpenoids such as germacrane and oplopane abrotanifolon derivatives and lupane-type tri-terpenoids [12,13,14]. Triterpenes comprise one of the most interesting groups of natural products due to their high potential as pharmacological agents, including leishmanicidal, trypanocidal and antiplasmodial activity [15].

As part of our ongoing research on Saudi medicinal plant metabolites with antiprotozoal potential, triterpenoid constituents from *K. odora* were isolated, structurally analyzed and evaluated for antiprotozoal potential against the protozoan parasites *Plasmodium falciparum*, *Leishmania infantum*, *Trypanosoma cruzi* and *T. brucei*. To assess selectivity, cytotoxicity was determined on MRC-5 fibroblasts.

## 2. Results and Discussion

### 2.1. Phytochemistry

Compound **1** was isolated as a white powder. Its ^13^C-NMR and DEPT spectrum (Table 1) exhibited 30 carbons, including seven singlet methyl groups, eleven methylenes, six methines one of which is oxygenated, and six quaternary carbons, which taken together revealed the basic skeleton of a pentacyclic triterpene. NMR spectra include signals for isopropenyl group (olefinic quaternary carbons at δ_c_ 150.9, methylene carbon at δ_c_ 109.3, protons singlets at δ_H_ 4.69 and 4.57, and methyl singlet at δ_H_ 1.64) suggesting lupane type triterpene. The ^1^H-NMR spectrum (Table 1) showed one oxymethine proton at C-3 as a broad singlet (δ_H_ 3.39). The proton signal at *δ*_H_ 2.38 (1H, *td*, 11.0, 10.0, 5.5 Hz) further revealed a typical H-19*β* lupane structure [16]. By comparing these NMR data with previously published data [17], compound **1** was characterized as epilupeol.

Compound **2** was also isolated as white crystals. The NMR data (Table 1) suggested a lupane skeleton. The NMR data were identical with those of epilupeol, except for the ring A signals. Compound **2** displayed a double doublet at δ_H_ 4.47 confirming the α-orientation of the C-3 proton [18]. It also showed additional signals attributed to the presence of an acetoxy group (carbonyl signal at δ_C_ 171.0 and methyl signals at δ_C_ 27.9 and δ_H_ 2.04). By comparison with previously reported data [19], compound **2** was identified as lupeol acetate.

Compound **3** was isolated as a white powder. The ^1^H- and ^13^C-NMR spectra (Table 1) revealed the presence of five tertiary methyls, two secondary methyls, nine methylenes, seven methines, one of which was oxygenated, and six quaternary carbons, which indicated a pentacyclic triterpenoid. The NMR spectrum showed a double bond (δ_C_ 124.5, 138.2 ppm) and a carboxyl group (δ_C_ 178.6) signals. The positions of the carboxy group and double bond were confirmed by a HMBC correlation study. By comparison with previously reported data [20] Compound **3** was identified as ursolic acid (3β-hydroxy-urs-12-en-28-oic acid).

The proton and carbon signals in the ^1^H- and ^13^C-NMR spectra (Table 1) of Compound **4** were very similar to those of compound 3, except for the signals of ring D. The carboxy group signals in the NMR spectrum disappeared and it showed one additional oxymethine signal (δ_H_ 4.19; δ_C_ 67.4) and an additional methyl singlet at δ_H_ 0.80. The hydroxyl and methyl groups were positioned at C-16 and C-17 based on a HMBC correlation study. Comparing the spectral data with those reported in the literature [21], Compound **4** was identified as brein (urs-12-ene-3β,16β-diol).

The ^13^C-NMR data of Compound **5** (Table 1) were very similar to those of Compound **4** and differed in the signals for rings C and D. This was attributed to the presence of an α-hydroxy at C-11 and the disappearance of the β-hydroxyl at position 16. Spectral data of **5** are reported for the first time based on DEPT, HMBC and HSQC evaluation and comparison of NMR data of structurally related compounds [21] and the published ^1^H-NMR data of its acetate form [22]. Compound **5** was identified as 3β, 11α-dihydroxy urs-12-ene.

**Figure 1:**
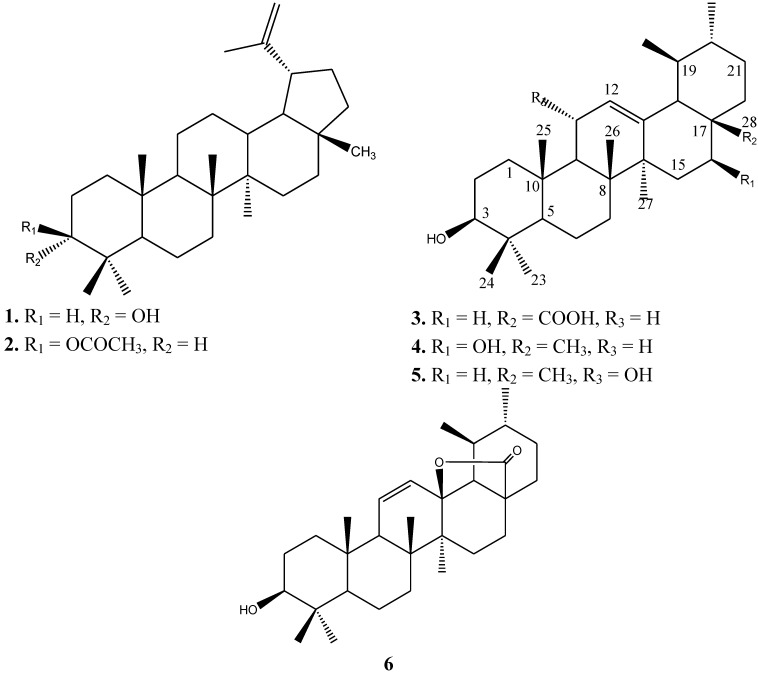
Structures of the triterpenoid compounds **1**–**6**.

The NMR spectral data of Compound **6** (Table 1) exhibited characteristic signals of an oleanan-28,13β-olide. The ^13^C-NMR spectrum displayed resonances for a double bond at δ_C_ 129.9 and 135.0 for C-11 and C-12, in addition to an oxygenated quaternary carbon at 91.9 and a carbonyl at 182.6 for C-13 and C-28, respectively. These data confirmed the olean-11-en,28,13β-olide structure. These results are in agreement with previously reported [23] data for ursolic acid lactone **6**. Compounds **1** and **3**–**6** were isolated for the first time from *K. odora.* To the best of our knowledge, this is also the first report on the occurrence of ursane type triterpenes in the genus *Kleinia.* The structures are summarized in Figure 1.

### 2.2. Antiprotozoal Activity

Petroleum ether and chloroform extracts of *K. odora* exhibited potent activity against *T. brucei* with IC_50_ values of 0.5 µg/mL (Table 2). The chloroform extract gave slightly higher selectivity (SI = 63) compared to the petroleum ether extract (SI = 39). The chloroform extract also displayed moderate activity against *P. falciparum* schizonts and intracellular amastigotes of *L. infantum* (IC_50_ 8 µg/mL). In comparison, the petroleum ether extract showed similar activity against *P. falciparum*, *L. infantum* and *T. cruzi* (IC_50_ 8.6, 6.8 and 5.7 µg/mL) but with lower selectivity. This motivated us to investigate the antiprotozoal activity of the isolated and identified triterpenoids from the chloroform extract.

Brein (**4**) and 3β, 11α-dihydroxy urs-12-ene (**5**) were found to be active and selective against more than one of the investigated protozoa. Brein (**4**) showed selective and potent activity against *T. brucei* (IC_50_ 2.3 µM, SI >27.8), with moderate side-activity against *P. falciparum*, *L. infantum* and *T. cruzi* (IC_50_ 9.3–9.9 µM) and acceptable selectivity (SI > 6.5). Compound **5** showed potent and selective activity against *L. infantum* (IC_50_ 3.2 µM, SI > 20) with some side-activity against *T. cruzi* and *T. brucei* (IC_50_ 8.1 and 7.9 µM, respectively, SI > 7.9).

Although ursolic acid (**3**) showed efficacy against *T. brucei* (IC_50_ 2.2 µM) comparable to that of Brein (**4**), its effect against *T. cruzi* and *L. infantum* (IC_50_ 8.8 and 7.4 µM) was associated with low selectivity. Meanwhile, ursolic acid lactone **6** was devoid of any antiprotozoal activity at the highest concentration tested (64 µg/mL). The antitrypanosomal and antileishmanial activity of urosolic acid obtained herein is in consistent with the data found in literature. It has been shown to be active against *T. brucei* at IC_50_ 1.0 ± 0.2 µg/mL (2.2 µM) [24,25]. In addition, it was also claimed active against *T. cruzi* with IC_50_ 21 µM [26] and *L. donovani* (IC_50_ = 3.5 µg/mL) (7.6 µM) [27]. It is worthy of pointing out that in our investigation, the antiplasmodial activity of this compound (IC_50_ 29.7 µM) is not in agreement with previously reported data which lists IC_50_ values of 3.1 µg/mL (6.8 µM) [28] and 4.9 µg/mL (10.7 µM) [29].

**Table 1:** ^1^H- and ^13^C-NMR data for compounds **1**–**6**.

#	Compound 1 in CDCl_3_		Compound 2 in CDCl_3_		Compound 3 in DMSO		Compound 4 in MeOD		Compound 5 in CDCl_3_		Compound 6 in MeOD	
	δ_C_	δ_H_	δ_C_	δ_H_	δ_C_	δ_H_	δ_C_	δ_H_	δ_C_	δ_H_	δ_C_	δ_H_
**1**	33.2		38.4		38.2		40.1		39.7		39.4	
**2**	25.1		21.3		28.2		27.9		27.4		27.7	
**3**	76.3	3.39 (*br s*)	81.0	4.47 (*dd*, 5.5, 10.5 Hz)	76.8		79.7	3.18 (*d*, 7.5 Hz)	78.8	3.23 (*br s*)	79.5	3.2 (*dd*, 6.5, 10 Hz)
**4**	37.5		38.0		38.8		39.9		39.1		40.0	
**5**	49.0		55.4		54.8		56.7		55.3		56.1	
**6**	18.3		18.2		18.0		19.5		18.4		18.8	
**7**	34.1		34.2		32.7		31.8		33.5		32.3	
**8**	41.0		40.8		40.0		41.0		43.3		41.5	
**9**	50.1		50.3		47.1		48.5		55.8	2.2 (*d*, 10 Hz)	54.0	
**10**	37.2		37.8		36.7		36.8		38.1		37.5	
**11**	20.8		20.9		22.9		24.2		68.3	4.26	129.9	5.61 (*d*, 8.5 Hz)
**12**	25.3		23.7		124.5	5.16 (br s)	126.3	5.23 (*br s*)	128.6	5.18 (*d*, 2 Hz)	135.0	6.07 (*d*, 10 Hz)
**13**	38.0		37.1		138.2		139.7		143.0		91.9	
**14**	42.9		42.8		41.6		45.1		42.1		42.0	
**15**	27.4		25.1		27.2		36.4		27.9		26.6	
**16**	35.6		35.6		25.6		67.4	4.19 (*d*, 6.5 Hz)	27.4		23.9	
**17**	43.0		43.0		46.7		39.5		33.6		46.6	
**18**	48.3		48.3		52.4	2.11 (*d*, 11.5 Hz)	62.4		58.0		61.8	
**19**	48.0	2.38 (*td*, 11, 10, 5.5 Hz)	48.0	2.38 (*td*, 11, 10, 5.5 Hz)	38.4		40.9		39.3		41.5	
**20**	150.9		151.0		38.1		40.0		39.4		39.4	
**21**	29.8		29.8		30.4		31.8		31.1		32.3	
**22**	40.0		40.0		36.5		36.8		41.3		32.4	
**23**	28.3	0.82 (*s*)	27.4	0.85 (*s*)	21.1	0.90 (*s*)	28.8	0.82 (*s*)	28.7		28.3	0.96 (*s*)
**24**	22.2	0.93 (*s*)	16.5	0.84 (*s*)	16.9	0.68 (*s*)	16.3	1.02 (*s*)	23.1	1.29 (*s*)	15.6	0.80 (*s*)
**25**	16.0	0.84 (*s*)	16.2	1.05 (*s*)	15.1	0.87 (*s*)	16.4	1.02 (*s*)	16.8	0.90 (*s*)	19.4	1.05 (*s*)
**26**	15.9	1.03 (*s*)	16.0	0.83 (*s*)	16.9	0.76 (*s*)	18.3	1.10 (*s*)	18.0	1.26 (*s*)	18.4	0.98 (*s*)
**27**	14.6	0.96 (*s*)	14.5	0.79 (*s*)	23.3	1.10 (*s*)	25.1	1.20 (*s*)	23.1	1.30 (*s*)	16.6	1.25 (*s*)
**28**	18.0	0.78 (*s*)	18.0	0.94 (*s*)	178.6		22.1	0.80 (*s*)	28.2	1.10 (*s*)	182.6	-
**29**	109.3	4.69 (br *s*), 4.57 (br *s*)	109.3	4.69 (*br s*), 4.57 (*br s*)	16.9	0.82, (*d*, 6.5 Hz)	17.0	0.84 (*d*, 6.5 Hz)	17.6	0.90 (*d*)	18.3	1.05 (*d*, 7.5 Hz)
**30**	19.3	1.64	19.3	1.68 (*s*)	21.1	0.92, (*d*, 7.0 Hz)	21.1	0.97 (*d*)	21.4	0.95 (*d*)	19.6	0.98 (*d*, 6.0 Hz)
**1’**			171.0									
**2’**			27.9	2.04 (*s*)								

**Table 2:** Antiprotozoal activity and cytotoxicity of triterpenoid constituents isolated from the plant *K. odora.*

Sample	*P. falciparum*		*L. infantum*		*T. cruzi*		*T. brucei*		MRC-5
	IC_50_	*SI*	IC_50_	*SI*	IC_50_	*SI*	IC_50_	*SI*	IC_50_
**Petroleum ether extract**	8.6 ± 2.1	*2.3*	6.8 ± 1.6	*3*	5.7 ± 1.6	*3.4*	0.5 ± 0.1	*39*	19.4 ± 3.4
**Chloroform extract**	8.2 ± 1.9	*4*	8.1 ± 2.3	*4*	31.0 ± 4.9	*-*	0.5 ± 0.1	*63*	31.3 ± 4.2
**Cmpd. 3 **	29.7 ± 5.9	*<1*	7.4 ±1.9	*1.5*	8.8 ± 2.3	*1.3*	2.2 ± 0.6	*5.2*	11.4 ± 2.1
**Cmpd. 4 **	9.7 ± 3.2	*>6.6*	9.3 ± 2.2	*>6.9*	9.9 ± 2.6	*>6.5*	2.3 ± 0.4	*>27.8*	>64.0
**Cmpd. 5 **	23.9 ± 5.7	*2.7*	3.2 ± 0.9	*>20*	8.1 ± 1.8	*>7.9*	7.8 ± 1.8	*>8.2*	>64.0
**Cmpd. 6 **	>64.0	-	>64.0	-	>64.0	-	40.9 ± 8.1	*-*	>64.0
**Chloroquine**	**0.3 ± 0.05**								
**Miltefosine**			**10.4 ± 2.1**						
**Benznidazole**					**1.9 ± 0.3**				
**Suramine**							**0.03 ± 0.01**		
**Tamoxifen**									**11.4 ± 3.2**

IC50 μg/mL for extracts; µM for pure compounds.

These divergent results may be attributed to the diversity of parasite strains, parasite load, stages of parasite life cycle or be related to differences in the experimental conditions [30]. Ursolic acid lactone **6** was reported to have activity against *L. donovani* (IC_50_ 191.52 µM) without cytotoxicity towards peripheral blood mononuclear cells [31]. In our opinion, this result was misinterpreted by the authors since the standard miltefosine under the same experimental conditions showed an IC_50_ of 9.31 µM [31]; hence, the compound should be considered as inactive. To the best of our knowledge, this is the first report on the antiprotozoal evaluation of compounds **4**, **5** and **6** against *P. falciparum*, *L. infantum*, *T. cruzi*, and *T. brucei*.

## 3. Experimental

### 3.1. General

The 1D-NMR and 2D-NMR spectra were recorded on a Bruker AMX-400 spectrometer with tetramethylsilane (TMS) as an internal standard. Thin layer chromatography (TLC) was performed on precoated silicagel F254 plates (E. Merck, Darmstadt, Germany). All chemicals were purchased from Sigma Chemical Company (St. Louis, MO, USA).

### 3.2. Plant Materials

The plant *Kleinia odora* was collected from the South of Saudi Arabia in February 2011 and identified at the Pharmacognosy Department, College of Pharmacy, King Saud University. A voucher specimen was deposited at the Pharmacognosy Department, College of Pharmacy, King Saud University (Voucher # P-15129).

### 3.3. Extraction and Isolation

The air-dried and powdered aerial part of *K. odora* (1 kg) was extracted by maceration with 70% ethanol (4 × 2 L) at room temperature. The combined obtained ethanolic extract was filtered and evaporated at 40 °C using a rotary evaporator. The dried ethanolic extract (50 g) was subsequently redissolved in water (200 mL) and partitioned successively for several times with petroleum ether (3 × 200 mL), chloroform (3 × 200 mL) and *n*-butanol (3 × 200 mL) to provide the corresponding extracts. The petroleum ether extract (6 g) was subjected to column chromatography on pre-packed silica gel columns (35 mm i.d. × 350 mm) to give nine fractions. The elution was performed with a gradient of hexane-ethyl acetate (10:1) to pure ethyl acetate. TLC analysis of the fractions with anisaldehyde/sulfuric acid and heating at 100 °C allowed the analysis of the nine fractions. Fraction 1 was further purified by using a chromatotron (Harrison Research, Palo Alto, CA, USA) (silica gel 60 F_254_, layer thickness 2 mm, Merck, Darmstadt, Germany) to give Compound **2** (27 mg). The elution was performed with a mobile phase composed of hexane–dichloromethane (60:40, v/v). The chloroform extract (4 g) was applied on a silica gel column and eluted with a gradient of dichloromethane-ethyl acetate (9:1) to pure ethyl acetate to give seven fractions. Fraction 1 was rechromatographed on a silica gel column (dichloromethane-acetone, 9:1) and on a RP-18 column (MeOH–H_2_O, 90:10) to produce Compound **4** (48 mg) and Compound **6** (21 mg). Direct crystallization of fraction 2 eluted by 30% acetone-dichlormethane gave Compound **3** (35 mg). Fraction 5 was purified on a RP-18 column (MeOH–H_2_O, 90:10) to give Compound **5** (8 mg). Fraction 7 (80 mg) was subjected to RP-18 column chromatography with MeOH–H_2_O (90:10) as a solvent to produce Compound **1** (20 mg).

*Epilupeol* (**1**). Amorphous powder; m.p. 205 °C; NMR (CDCl_3_): see Table 1.

*Lupeol acetate* (**2**). White crystals; m.p. 215–218 °C; NMR (DMSO): see Table 1.

*Ursolic acid* (**3**). White powder; m.p. 236 °C; NMR (DMSO): see Table 1. 

*Urs-12-ene-3β,16β-diol* (**4**). Solid; m.p. 221 °C; NMR (MeOD): see Table 1.

*3β,11α-Dihydroxyurs-12-ene* (**5**). Amorphous powder; NMR (CDCl_3_): see Table 1. 

*3-Hydroxy-13,28-epoxyurs-11-en-28-one* (**6**, *ursolic acid lactone*)*.* White powder; m.p. 268 °C; NMR (MeOD): see Table 1.

### 3.4. Antiprotozoal Assay

#### 3.4.1. Standard Drugs

For the different tests, appropriate reference drugs were used as positive control: tamoxifen for MRC-5, chloroquine for *P. falciparum*, miltefosine for *L. infantum*, benznidazole for *T. cruzi* and suramin for *T. brucei*. All reference drugs were either obtained from the fine chemical supplier Sigma-Aldrich, Taufkirchen, Germany (tamoxifen, suramin) or from WHO-TDR, Geneva, Switzerland (chloroquine, miltefosine, benznidazole).

#### 3.4.2. Biological Assays

The integrated panel of microbial screens and standard screening methodologies were adopted as previously described [32]. All assays were performed in triplicate at the Laboratory of Microbiology, Parasitology and Hygiene at the University of Antwerp (Belgium). Plant extracts were tested at 5 concentrations (64, 16, 4, 1 and 0.25 μg/mL) to establish a full dose-titration and determination of the IC50 (inhibitory concentration 50%). The final in-test concentration of DMSO did not exceed 0.5%, which is known not to interfere with the different assays [32]. Selectivity of activity was assessed by simultaneous evaluation of cytotoxicity on a fibroblast (MRC-5) cell line. The criterion for activity was an IC50 <10 μg/mL and a selectivity index (SI) of > 4.

#### 3.4.3. Antiplasmodial Activity

Chloroquine-resistant *P. falciparum* K 1-strain was cultured in human erythrocytes O^+^ at 37 °C under a low oxygen atmosphere (3% O2, 4% CO2, and 93% N2) in RPMI-1640, supplemented with 10% human serum. Infected human red blood cells (200 μL, 1% parasitaemia, 2% haematocrit) were added to each well and incubated for 72 h. After incubation, test plates were frozen at −20 °C. Parasite multiplication was measured using the Malstat assay, a colorimetric method based on the reduction of 3-acetyl pyridine adenine dinucleotide (APAD) by parasite-specific lactate-dehydrogenase (pLDH) [32,33].

#### 3.4.4. Antileishmanial Activity

*L. infantum* MHOM/MA(BE)/67 amastigotes were collected from the spleen of an infected donor hamster and used to infect primary peritoneal mouse macrophages. To determine *in vitro* antileishmanial activity, 3 × 10^4^ macrophages were seeded in each well of a 96-well plate. After 2 days outgrowth, 5 × 10^5^ amastigotes/well, were added and incubated for 2 h at 37 °C. Pre-diluted plant extracts were subsequently added and the plates were further incubated for 5 days at 37 °C and 5% CO_2_. Parasite burdens (mean number of amastigotes/macrophage) were microscopically assessed on 500 cells after Giemsa staining of the testplates, and expressed as a percentage of the blank controls without plant extract.

#### 3.4.5. Antitrypanosomal Activity

*Trypanosoma brucei* Squib-427 strain (suramin-sensitive) was cultured at 37 °C and 5% CO_2_ in Hirumi-9 medium [34], supplemented with 10% fetal calf serum (FCS). About 1.5 × 10^4^ trypomastigotes/well were added to each well and parasite growth was assessed after 72 h at 37 °C by adding resazurin [35]. For Chagas disease, *T. cruzi* Tulahuen CL2 (benznidazole-sensitive) was maintained on MRC-5 cells in minimal essential medium (MEM) supplemented with 20 mM l-glutamine, 16.5 mM sodium hydrogen carbonate and 5% FCS. In the assay, 4 × 10^3^ MRC-5 cells and 4 × 10^4^ parasites were added to each well and after incubation at 37 °C for 7 days, Parasite growth was assessed by adding the alpha-galactosidase substrate chlorophenol red alpha-D-galactopyranoside [36]. The color reaction was read at 540 nm after 4 h and absorbance values were expressed as a percentage of the blank controls.

#### 3.4.6. Cytotoxicity against MRC-5 Cells

MRC-5 SV2 cells were cultivated in MEM, supplemented with l-glutamine (20 mM), 16.5 mM sodium hydrogen carbonate and 5% FCS. For the assay, 10^4^ MRC-5 cells/well were seeded onto the test plates containing the pre-diluted sample and incubated at 37 °C and 5% CO_2_ for 72 h. Cell viability was assessed fluorimetrically after 4 h of addition of resazurin. Fluorescence was measured (excitation 550 nm, emission 590 nm) and the results were expressed as % reduction in cell viability compared to control.

## 4. Conclusions

Two lupane and four ursane triterpenes were from isolated aerial parts of *K. odora* and identified. Ursane type triterpenes from the biologically active chloroform extract were investigated for their antiprotozoal potential. All were found to have activity against more than one type of the tested parasites, with exception of compound **6**.
